# CONTEXTUALIZING HOW DAILY DISCRIMINATION EXPERIENCES IMPACT EVERYDAY METAMEMORY

**DOI:** 10.1093/geroni/igad104.3717

**Published:** 2023-12-21

**Authors:** Timothy Ly, Rebecca Allen, Jeanne Cundiff, Jason DeCaro

**Affiliations:** The University of Alabama, Tuscaloosa, Alabama, United States; The University of Alabama, Tuscaloosa, Alabama, United States; The University of Alabama, Tuscaloosa, Alabama, United States; The University of Alabama, Tuscaloosa, Alabama, United States

## Abstract

Examining day-to-day responses to everyday stressors, such as discrimination, contextualizes how cognitive aging trajectories may vary across the lifespan. Furthermore, recent research investigating stress-induced impairment of metamemory may address the relationship between discrimination experiences and cognitive impairment. This study sought to articulate the association between daily experiences of discrimination and impaired metamemory across the lifespan, as defined by the number of subjective cognitive complaints, using data collected from the Midlife in the United States Refresher Study Daily Diary Project (N = 782; ages 20-75; M = 47.91 years, SD = 12.67 years). A two-level linear mixed model was conducted to examine the within and between-persons relationships concerning lifetime and daily experiences of discrimination, daily affect balance, baseline objective cognitive performance, and sociodemographic variables (age, race, ethnicity, and sex) on impaired everyday metamemory. Results from linear mixed model analyses showed significant within-person fixed effects of daily discrimination and daily affect balance on metamemory accuracy, as well as significant between-persons fixed effects of race and ethnicity on metamemory accuracy. Furthermore, significant interactions were found between race and daily discrimination experiences, ethnicity and daily discrimination experiences, daily affect balance and daily discrimination experiences, daily affect balance and age, and endorsement of lifetime experiences of discrimination and daily discrimination experiences on day-to-day metamemory accuracy. These findings extend our understanding of how discrimination experiences may impair metacognitive processes and demonstrate the need for more research into understanding metamemory accuracy as an underlying mechanism by which the psychosocial stressor of discrimination impacts cognition across the lifespan.

